# Effects of seasonal changes on the carbon dynamics in mixed coniferous forests

**DOI:** 10.1371/journal.pone.0267365

**Published:** 2023-04-20

**Authors:** Tong Gao, Xinyu Song, Yunze Ren, Hui Liu, Yuan Meng, Xibin Dong

**Affiliations:** College of Engineering and Technology, Northeast Forestry University, Harbin, Heilongjiang, China; The Ohio State University, UNITED STATES

## Abstract

We investigated the residual rate and mass loss rate of litter, as well as the carbon release dynamics of litter and soil across seasons, to better understand the effects of seasonal fluctuations on carbon dynamics in mixed coniferous forests. The study was carried out in natural mixed coniferous forests in the Xiaoxinganling region of Heilongjiang Province, China, and the number of temperature cycles in the unfrozen season, freeze–thaw season, frozen season, and thaw season was controlled. The goal of the study was to examine how the carbon release dynamics of litter and soil respond to the freeze–thaw process and whether there are differences in carbon release dynamics under different seasons. Repeated-measures analysis of variance was used to analyze the residual mass rate and mass loss rate of litter, litter organic carbon and soil organic carbon during the unfrozen season, freeze-thaw season, frozen season, and thaw season. Litter decomposition was highest in the unfrozen season (15.9%~20.3%), and litter and soil carbon were sequestered throughout this process. Temperature swings above and below 0°C during the freeze–thaw season cause the litter to physically fragment and hasten its decomposition. Decomposition of litter was still feasible during the frozen season, and it was at its lowest during the thaw season (7.2%~7.8%), when its organic carbon was transported to the soil. Carbon migrates from undecomposed litter to semi-decomposed litter and then to soil. The carbon in the environment is fixed in the litter (11.3%~18.2%) and soil (34.4%~36.7%) in the unfrozen season, the carbon-fixing ability of the undecomposed litter in the freeze-thaw season is better, and the carbon in the semi-decomposed litter is mostly transferred to the soil; the carbon-fixing ability of the litter in the frozen season is worse (-3.9%~ -4.3%), and the organic carbon in the litter is gradually transferred to the soil. The carbon-fixing ability of the undecomposed litter in the thaw season is stronger, and the organic carbon in the semi-decomposed litter is mostly transferred to the soil. Both litter and soil can store carbon; however, from the unfrozen season until the thaw season, carbon is transported from undecomposed litter to semi-decomposed litter and to the soil over time.

## Introduction

Seasonal freeze–thaw cycles affect the majority of the Earth’s mid-latitudes [1]; at mid-to-high latitudes and high altitudes, these cycles have a substantial effect on forest ecosystem processes [2,3]. Seasonal freeze–thaw cycles can last up to 5–6 months per year in Northeast China, which is one of China’s primary freeze–thaw erosion areas. The freeze–thaw cycle is particularly active in Heilongjiang Province, which is located in China’s alpine region [4]. Forests play a key role in regulating the global biosphere’s carbon balance, as they are both an important carbon sink and source of atmospheric CO_2_ [5,6], and sustain a substantial carbon pool [7], with carbon stocks accounting for more than 80% of terrestrial vegetation carbon stocks [8]. Forest litter is the principal nutrient reservoir in forest ecosystems and is produced by the metabolism of forest plants during their growth and development [9], it is an important aspect of the material cycle of forest ecosystems and thus an important component of forest ecosystems [10]. The rate of litter decomposition and conversion reflects the rate of nutrient return and nutrient cycling in forests [11]. Physical damage, microbial activity, waterlogging, and their combined effect throughout the freeze–thaw season are the main causes of net mass loss and nutrient release during the decomposition process [12], and litter decomposition affects soil organic matter formation and nutrient release [13]. Warming [14,15], affects soil organic carbon through its effect on plant growth, which alters the number of plant residues returned to the soil, the pace of organic carbon decomposition, and the amount of organic carbon released from the soil [16]. Many studies have shown that climate change can affect litter decomposition [17,18], the amount of organic carbon released from litter [19,20], and the carbon cycle of entire ecosystems [21–23]. Litter has a major effect on the organic carbon in soil [24,25], but few studies have examined the carbon dynamics of mixed coniferous forests in seasonal freeze-thaw areas. In this study, we investigated whether there are differences in carbon release dynamics in different seasons and controlled the number of temperature cycles to (1) determine the effect of the number of temperature cycles on the decomposition level of litter, litter organic carbon, and soil organic carbon in the undecomposed layer and semi-decomposed layer; (2) clarify the response of residual mass rate, mass loss rate, and organic carbon, and soil organic carbon to seasonal changes under four seasons (non-freezing season, freeze-thawing season, freezing season, and thawing season); and (3) examine the process of carbon transport in different seasons.

## Materials and methods

### Overview of the study area

The experiment was conducted at Dongfanghong Forestry Field, Dailing Forestry Bureau, Xiaoxinganling Region, Heilongjiang Province, China, which is located 13.9 km southeast of Dailing District (46°50’8′′-47°21’32′′N, 128°37’46′′-129°17’50′′E). The site has an average slope of 10°, an average altitude of 600 m, and annual precipitation of 660 mm. The average annual temperature is 2.7°C. Summers are humid, cool, and wet, and winters are dry, cold, and snowy. The soil was mainly dark brown soil [26], it was found to contain 66.5 mg·kg^-1^ of available phosphorus, 61.3 mg·kg^-1^ of available potassium, 5.8 g·kg^-1^ of total nitrogen, 1.9 g·kg^-1^ of total phosphorus and 11.6 g·kg^-1^ of total potassium. The soil had a pH of 5.01 and composition of 42.1% sand, 31.1% silt, and 26.8% clay. The forest community type of the test site is a natural mixed coniferous-broad forest, and the main tree species are *Pinus koraiensis*, *Abies fabri*, *Picea asperata*, *Tilia tuan*, *Acer mono Maxim*, *Acer davidii Franch*, *Fraxinus mandshurica Rupr*, etc. Shrubs are mostly *Lonicera japonica*, *Acanthopanax senticosus*, *Spodiopogon cotulifer*. Herbaceous plants include *Scirpus planiculmis*, *Carex callitrichos*.

### Experimental procedures

On the basis of a whole-forest survey, a 100 m × 100 m sample plot was set up in a stand with a full range of tree species, a more uniform mix, and basically no human damage, three 30 m × 30 m quadrat were selected by diagonal sampling method [27,28]. In each season, three 1 m × 1 m litter samples were collected at random locations within each quadrat; litter and soil samples were collected separately. We collected the litter in the quadrat by hand. Then the litter was placed in plastic bags and brought back to laboratory for analysis. After collection, litter was allowed to dry naturally in the laboratory. The litter was collected from two layers: the undecomposed layer (fresh litter showing slight color change, an intact structure, and no symptoms of decomposition) and the semi-decomposed layer (darkened litter, damaged structure, and most litter decomposed). The litter on the soil surface was removed before soil samples were collected, and soil samples (length and width of 20 cm) were taken from the 0–10 cm depth.

Incubation boxes with 50.0 g of soil were established in the laboratory. In each quadrat, three replicate incubation boxes were built for each temperature cycle, and litter was placed on the soil surface for incubation. The amount of litter added to the three replicates in the incubator was 2.0 g, 3.0 g, and 4.0 g to minimize the effect of litter quality on organic carbon. The actual soil moisture content measured at each time point was used to calculate the soil moisture content. Temperature disparities were set for temperature cycling based on previously collected temperature data. Un-decomposed litter and semi-decomposed litter were placed separately in different incubation boxes. The amount of litter added to the experimental incubation boxes is shown in Table 1.

**Table 1:** Set-up of experimental incubator boxes and amount of litter addition.

Different degrees of decomposition of litter additions (g)	Season	Box	Number of temperature cycles						
			0	1	3	5	7	15	30
Undecomposed	Unfrozen 17~30°C	1	2.0						
	Freeze–thaw -5~5°C								
	2	3.0						
									Frozen -30~ -16°C
3	4.0						
								Thaw 5~ -5°C
Semi-decomposed	Unfrozen 17~30°C	1	2.0						
	Freeze–thaw -5~5°C								
	2	3.0						
									Frozen -30~ -16°C
3	4.0						
								Thaw 5~ -5°C

The temperature of the experimental sample site was monitored one year in advance, and four time periods were selected as representatives of the four seasons, respectively, using the average temperature of each time period for the experiment. Minimum and maximum temperatures of the unfrozen season (July), freeze–thaw season (October), frozen season (January), and thaw season (March) were 17°C and 30°C, -5°C and 5°C, -30°C and -16°C, and 5°C and -5°C, respectively. Each season was incubated with 30 temperature cycles, alternating between the lowest and highest temperatures for 12 h and a complete temperature cycle for 24 h. The boxes were removed at the 0th, 1st, 3rd, 5th, 7th, 15th, and 30th times of the temperature cycle, respectively. Separate cassettes were set up for each temperature cycle, totaling 168 cassettes for the four seasons, to prevent the samples in the cassettes from being damaged during sampling (4 seasons × 2 litter species × 7 freeze–thaws cycles × 3 replicates). The culture boxes were removed after the number of temperature cycles had been completed; the soil in the boxes was then air-dried and crushed through a 0.15-mm sieve, and the dead material was completely removed from the culture boxes. The samples were cleaned of surface soil particles and impurities, dried to constant weight, and weighed; they were then ground and passed through a 0.15-mm sieve prior to analysis. Table 2 shows the initial composition of the samples.

**Table 2:** Content of litter and initial soil fractions at different levels of decomposition during different seasons.

Season	Degree of decomposition of litter	Organic carbon from litter g/kg	Soil organic carbon g/kg
**Unfrozen**	Undecomposed	358.4±15.3	52.7±12.1
	Semi-decomposed	370.5±20.6	52.7±12.1
**Freeze–thaw**	Undecomposed	490.9±18.8	45.1±5.5
	Semi-decomposed	483.3±11.5	45.1±5.5
**Frozen**	Undecomposed	462.5±12.1	42.4±4.8
	Semi-decomposed	512.6±8.9	42.4±4.8
**Thaw**	Undecomposed	513.5±7.9	39.7±6.7
	Semi-decomposed	503.9±8.5	39.7±6.7

Note: Data in the table are mean ± standard deviation, n = 3.

### Data calculations

Residual rate of litter mass:

$$Q(\%)=M_{t}/M_{0}\times 100\%$$ (1)

Mass loss of litter at all stages:

$$L(\%)=(M_{t‐1}‐M_{t})/M_{0}\times 100\%$$ (2)

Rate of litter mass loss by stage (in counts):

$$R_{T}=(M_{t‐1}‐M_{t})/\Delta T$$ (3)

where *M*_0_ is the initial mass of the litter; *M*_*t*_ is the residual amount of litter at each sampling event; (*M*_*t -*1_—*M*_*t*_) is the difference in the residual amount of litter between two adjacent litter sampling events after drying and weighing (note: *M*_*t -*1_ is Mt measured in the previous period); and Δ*T* is the number of intervals between two adjacent sampling events.

### Analytical methods

We used potassium dichromate-concentrated sulphuric acid with thermal oxidation to determine the amount of litter and soil organic carbon [29,30]. Soil total nitrogen was measured using the Kjeldahl method [31]. Soil total phosphorus was measured spectrophotometrically after wet digestion with H_2_SO_4_ and HClO_4_ [32]. Soil fast-acting phosphorus is measured by sodium bicarbonate, soil total potassium is measured by NaOH-flame photometry, soil fast-acting potassium is measured by ammonium acetate-flame photometry [33]. Soil pH was measured with glass electrode in a 1: 2.5 soil/water suspension.

### Statistics analytical

All results were reported as means ± standard error (SE) for three replicates. Data analysis was conducted using IBM SPSS Statistics 26, and graphs were built using Origin 2019b. Repeated measurement ANOVA tested the significant differences in litter mass residual rate, litter organic carbon and soil organic carbon under the influence of season, litter decomposition, and temperatures. All significances mentioned in the text are at the 0.05 level, unless otherwise noted.

## Results

### Effect of seasonal changes on litter quality loss

The number of freeze–thaws had a significant effect (*P*<0.05) on the mass loss of litter (Table 3). In the unfrozen season, the mass residual rate of undecomposed litter under the 3rd and 5th freeze–thaw was significantly lower than the initial value; the mass residual rate of semi-decomposed litter under the 1st, 3rd, 5th, and 7th freeze–thaw was significantly lower than the initial value; and the mass residual rate under the 15th freeze–thaw was significantly lower than that under the 1st and 5th freeze–thaw. The mass residual rate of undecomposed litter was significantly lower than its initial value after the 3rd and 15th freeze–thaw in the freeze–thaw season, and the number of freeze–thaw treatments of semi-decomposed litter had no significant effect on the mass residual rate. The mass residual rate of undecomposed litter in the 5th and 15th freeze–thaw and semi-decomposed litter in the 3rd, 5th, and 15th freeze–thaw was much lower than initial values during the frozen season. The number of freeze–thaws that occurred throughout the thaw season had no effect on the bulk residue rate. In the unfrozen and frozen seasons, the rate of decrease in the quality of undecomposed litter was lower than that of semi-decomposed litter, and the rate of decrease in the quality of undecomposed litter was higher than that of semi-decomposed litter in the freeze–thaw season and thaw season.

**Table 3:** Results of repeated-measures ANOVA of litter mass loss at various degrees of decomposition as a function of various factors.

Factor	Degrees of Freedom	*F*	*P*
**Season**	3	0.048	0.986
**Degree of decomposition**	1	0.000	0.993
**Number of freeze–thaws**	6	21.987	<0.001**
**Season × Degree of decomposition**	3	0.008	0.999
**Number of freeze–thaws × Season**	18	0.908	0.540
**Number of freeze–thaws × Degree of decomposition**	6	0.516	0.159
**Number of freeze–thaws × Season × Degree of decomposition**	18	0.949	0.478

**, *P*<0.01.

**Fig 1:**
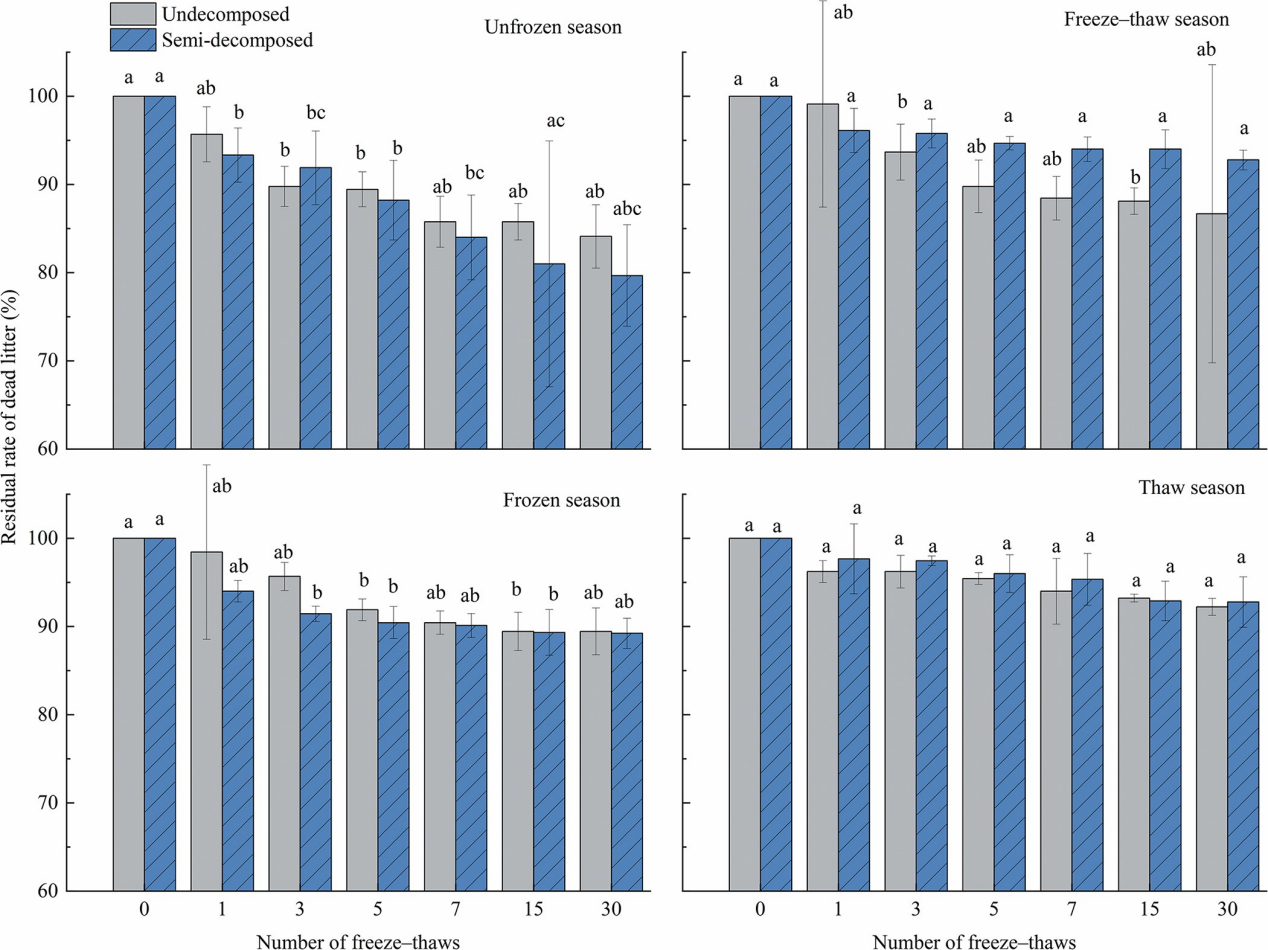
Effects of freeze–thaws in various seasons on the residual rate of litter at various levels of decomposition. Mean ± SE is shown in the figures. Different letters indicate significant difference at 0.05 level.

Fig 1 shows that the rate of decrease in undecomposed litter mass residues from initial values was highest in the unfrozen season (15.9%), followed by the freeze–thaw season (13.3%), frozen season (10.6%), and thaw season (7.8%); the rate of decrease in semi-decomposed litter mass residues from initial values was highest in the unfrozen season (20.3%), frozen season (10.8%), freeze–thaw season (7.2%), and thaw season (7.2%).

**Fig 2:**
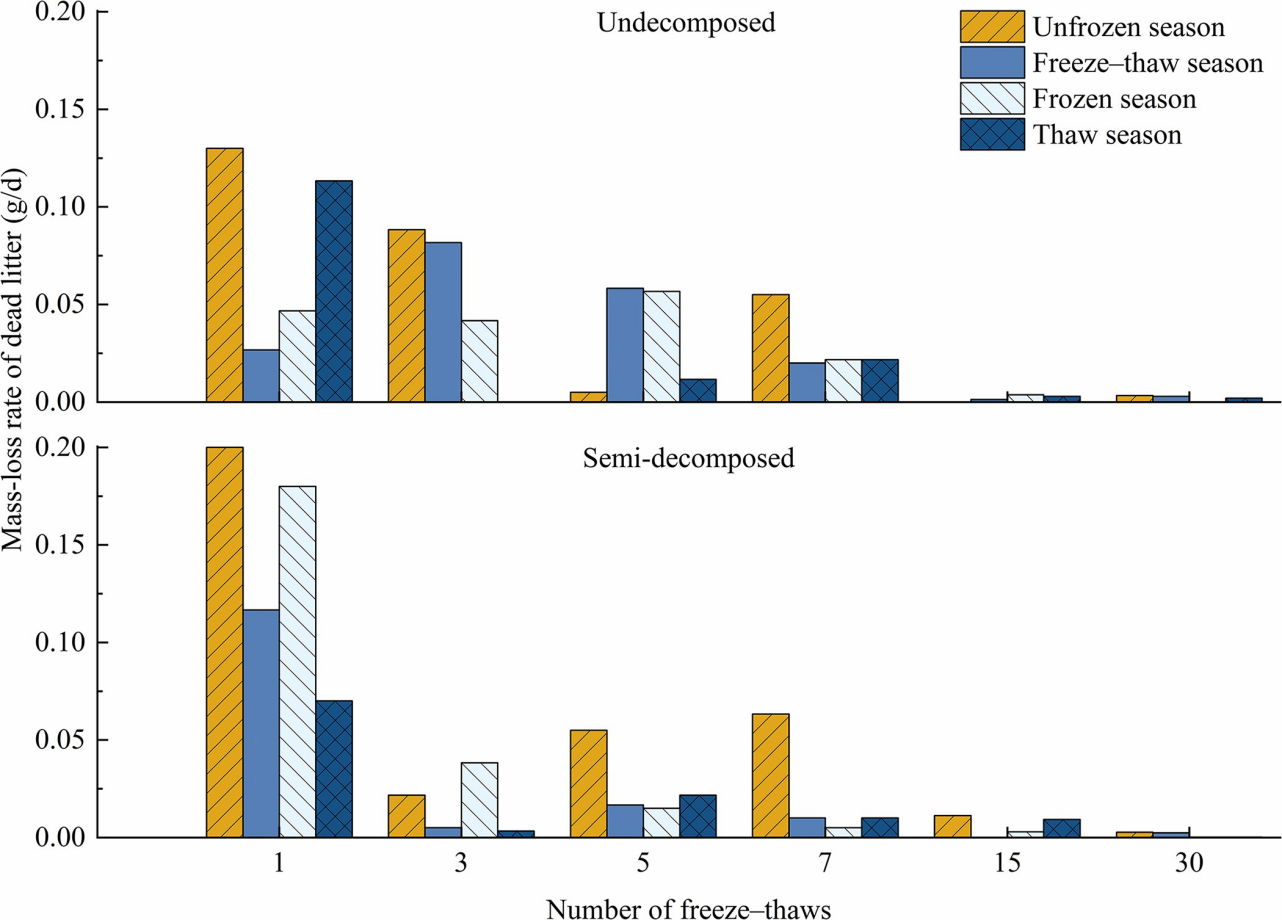
Effect of season on litter mass-loss rates at various levels of decomposition under freeze–thaw treatment.

Fig 2 shows that the overall rate of litter mass loss was higher in the unfrozen season than in the other three seasons. The rate of mass loss of litter first decreased, increased, and then decreased as the number of freeze–thaw cycles increased in the freeze–thaw season and thaw season. The rate of mass loss of undecomposed litter in the freeze–thaw seasons first increased and then decreased, and the rate of mass loss of semi-decomposed litter first decreased, increased, and then decreased; the rate of mass loss of semi-decomposed litter was much higher after the first freeze–thaw than after other freeze–thaws. During the frozen season, the rate of mass loss of undecomposed litter increased and then decreased, and the rate of semi-decomposed litter gradually decreased.

### Effect of seasonal changes on organic carbon dynamics in litter

**Fig 3:**
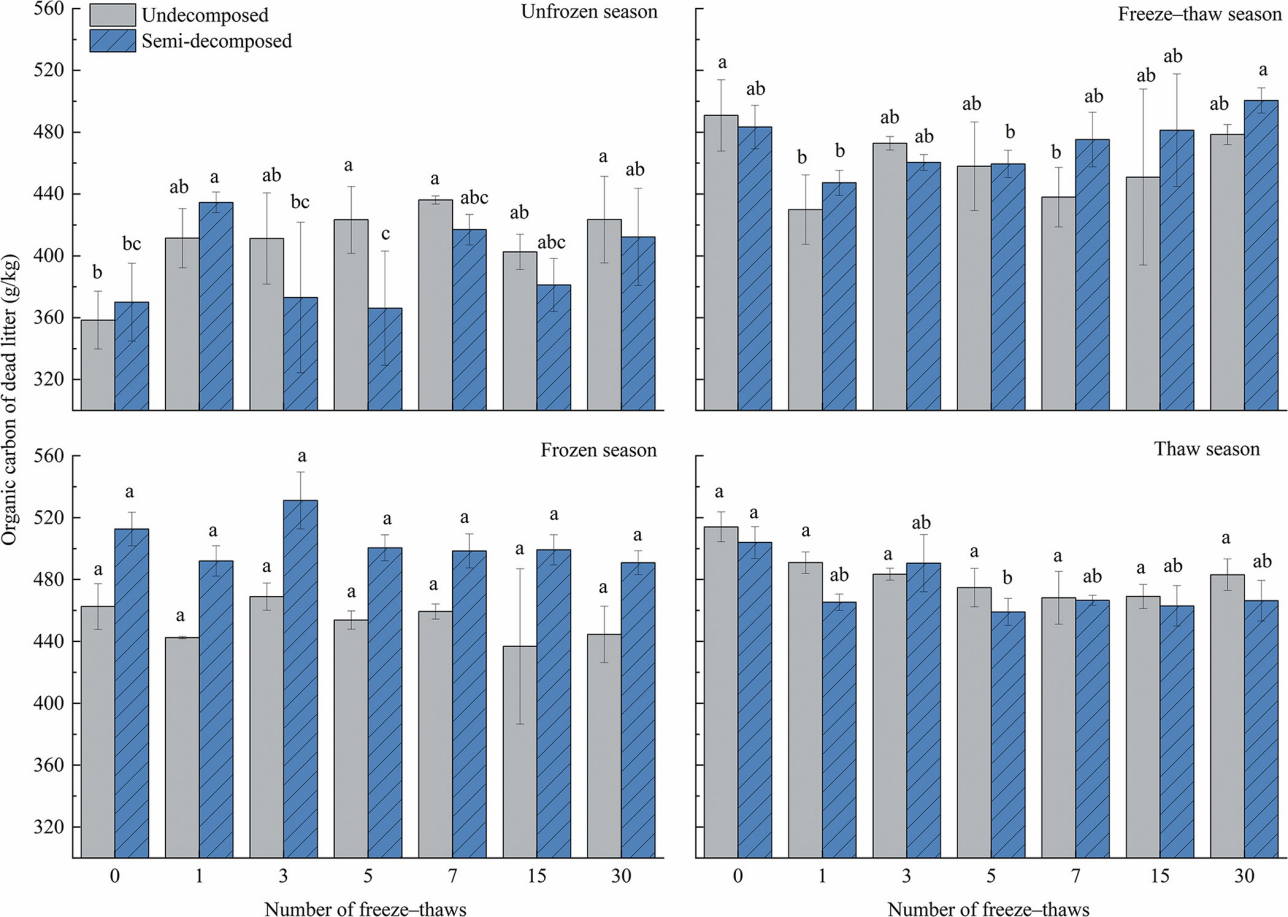
Effects of freeze–thaw time in various seasons on the carbon content of litter at various levels of decomposition. Mean ± SE is shown in the figures. Different letters indicate significant difference at 0.05 level.

Table 4 shows that the number of freeze–thaws and season had a significant effect (*P*<0. 05) on the organic carbon of litter. Fig 3 shows that the organic carbon content of litter increased in the unfrozen season and decreased in the frozen and thaw seasons. The organic carbon of undecomposed litter was lower than the initial value in the freeze–thaw season, and the opposite was the case for semi-decomposed litter after 30 freeze–thaws. The interaction between the decomposition degree of litter and season had a significant effect on litter organic carbon (*P* = 0.001). Changes in the organic carbon of litter at different decomposition levels varied after 30 freeze–thaw cycles. The organic carbon of undecomposed and semi-decomposed litter increased by 18.2% and 11.3% during the unfrozen season, respectively, and the organic carbon of undecomposed and semi-decomposed litter decreased and increased by 2.5% and 3.6% during the freeze–thaw season, respectively. The organic carbon of undecomposed litter and semi-decomposed litter decreased by 3.9% and 4.3%, respectively, during the frozen season. The organic carbon of undecomposed litter and semi-decomposed litter decreased by 5.9% and 7.5%, respectively, during the thaw season. During the frozen season, the organic carbon of semi-decomposed litter was substantially higher than that of undecomposed litter; specifically, the organic carbon of undecomposed litter was 10.1% lower than that of semi-decomposed litter.

**Table 4:** Seasonal variation in the organic carbon of litter at various levels of decomposition according to repeated-measures ANOVA.

Factor	Degrees of Freedom	*F*	*P*
**Degree of decomposition**	1	3.685	0.073
**Season**	3	55.682	< 0.001**
**Number of freeze–thaws**	6	5.003	0.010*
**Degree of decomposition × season**	3	9.417	0.001**
**Number of freeze–thaws × Degree of decomposition**	6	0.341	0.500
**Number of freeze–thaws × Season**	18	1.953	< 0.001**
**Number of freeze–thaws × Degree of decomposition × Season**	18	1.113	0.255

*, *P*<0.05

**, *P*<0.01.

Fig 3 shows that the number of freeze–thaws had a significant effect on litter organic carbon (*P* = 0.01). The interaction of freeze–thaw number and season had a significant effect on litter organic carbon (*P*<0.001). In the unfrozen season, the organic carbon of undecomposed litter after the 5th, 7th, and 30th freeze–thaws was significantly higher than the initial values, and the organic carbon of semi-decomposed litter was significantly higher for the 1st freeze–thaw than the 0th, 3rd, and 5th freeze–thaws. Organic carbon was significantly higher after the 30th freeze–thaw than after the 3rd freeze–thaw. In the freeze–thaw season, the organic carbon of undecomposed litter was significantly lower after the 1st and 7th freeze–thaws than initial values; the organic carbon of semi-decomposed litter was also higher after the 30th freeze–thaw than after the 1st and 5th freeze–thaws. The frequency of freeze–thaw cycles during the frozen season had no effect on the organic carbon content of litter. The organic carbon of semi-decomposed litter was significantly lower after the 5th freeze–thaw in the thaw season compared with the initial value, and the number of freeze–thaws had no effect on the organic carbon of undecomposed litter.

**Fig 4:**
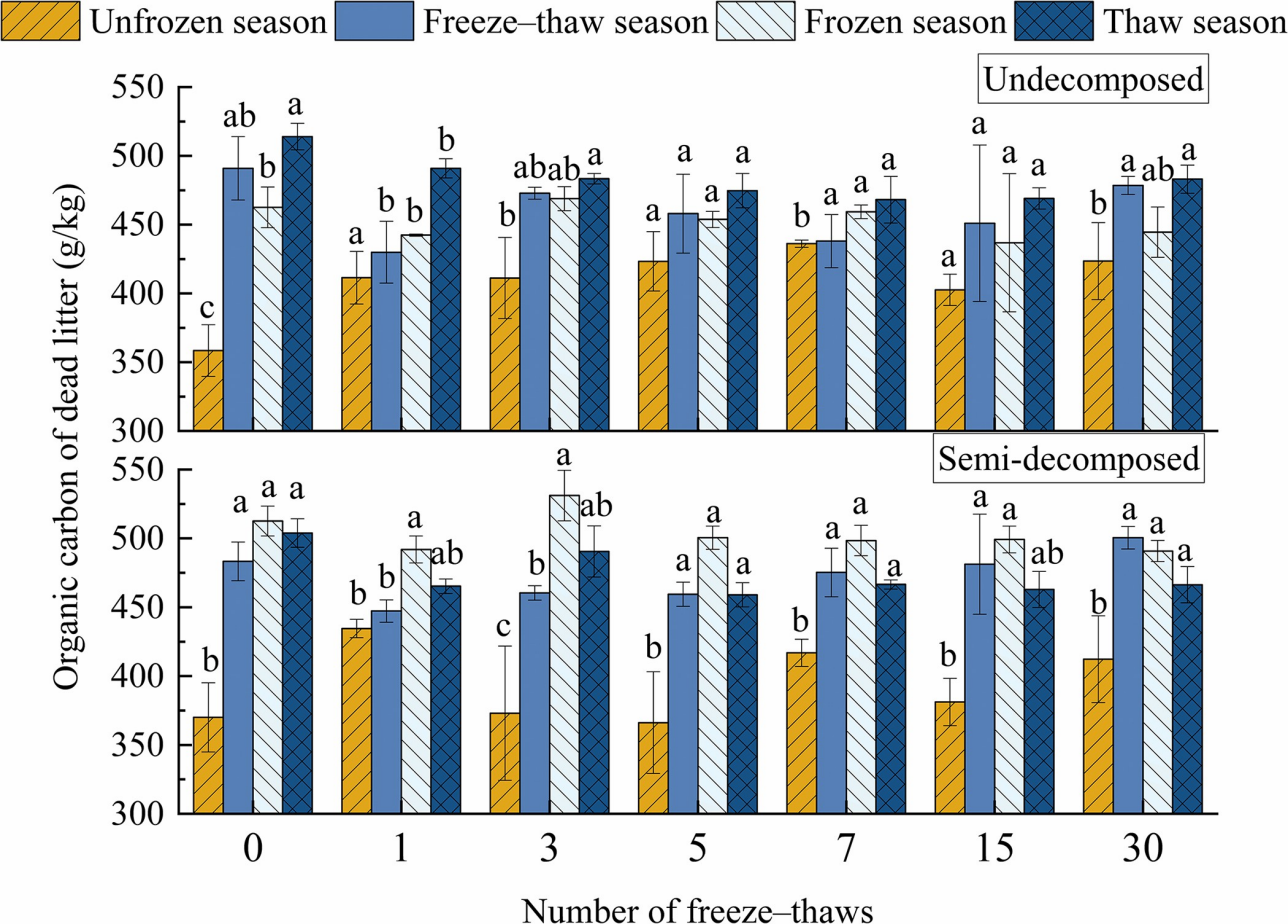
Effects of season on the carbon content of litter at various levels of decomposition under freeze–thaw treatment. Mean ± SE is shown in the figures. Different letters indicate significant difference at 0.05 level.

Organic carbon in the litter was significantly affected by season (*P*<0.001). The organic carbon of undecomposed litter was highest in the thaw season, followed by the frozen season and unfrozen season; the organic carbon of semi-decomposed litter was highest in the frozen season, followed by the thaw season and unfrozen season (Fig 4). During the freeze–thaw season, the organic carbon of undecomposed litter decreased to a level below that of the frozen season; it then increased to a level above that of the frozen season but below that of the thaw season. By contrast, the organic carbon of semi-decomposed litter initially decreased to a level below that of the frozen season and the thaw season; it then increased to a level above that of the frozen season and thaw season. The mean organic carbon of undecomposed litter in the thaw season was 6.8% and 18.0% higher than that in the frozen and unfrozen seasons, respectively. The mean organic carbon of semi-decomposed litter in the thaw and unfrozen seasons was 6.3% and 28.0% lower, respectively, than that in the frozen season. The organic carbon of semi-decomposed litter in the freeze–thaw season was initially lower than that in the thaw season and the frozen season. The organic carbon of semi-decomposed litter increased from lower than 4.3% and 6.1% to greater than 6.8% and 1.9% in the thaw and frozen seasons, respectively.

### Effect of seasonal changes on soil organic carbon

The effect of season and number of freeze–thaws on soil organic carbon in litter was highly significant (*P*<0.01) (Table 5). The soil organic carbon of undecomposed litter was greater than the initial value during the freeze–thaw season after 30 freeze–thaws, and the opposite pattern was observed for semi-decomposed litter; the soil organic carbon of litter was lower in the freeze–thaw season compared with the other seasons. The soil organic carbon of the unfrozen and semi-decomposed layers of litter increased by 36.4% and 36.7%, respectively; the organic carbon in undecomposed litter soil decreased by 29.2%, and the organic carbon in semi-decomposed litter soil increased by 2.2%. During the frozen season, soil organic carbon in the undecomposed and semi-decomposed layers increased by 13.2% and 23.6%, respectively; during the thaw season, soil organic carbon in the undecomposed and semi-decomposed layers of litter increased by 9.7% and 25.4%, respectively.

**Table 5:** Seasonal variation in the soil organic carbon in the litter at various states of decomposition was studied using repeated-measures ANOVA.

Factor	Degrees of Freedom	*F*	*P*
**Degree of decomposition**	1	0.133	0.720
**Season**	3	7.271	0.003**
**Number of freeze–thaws**	6	7.319	< 0.001**
**Degree of decomposition × season**	3	0.096	0.961
**Number of freeze–thaws × Degree of decomposition**	6	1.432	0.210
**Number of freeze–thaws × Season**	18	3.271	<0.001**
**Number of freeze–thaws × Degree of decomposition × Season**	18	0.768	0.732

**, *P*<0.01.

**Fig 5:**
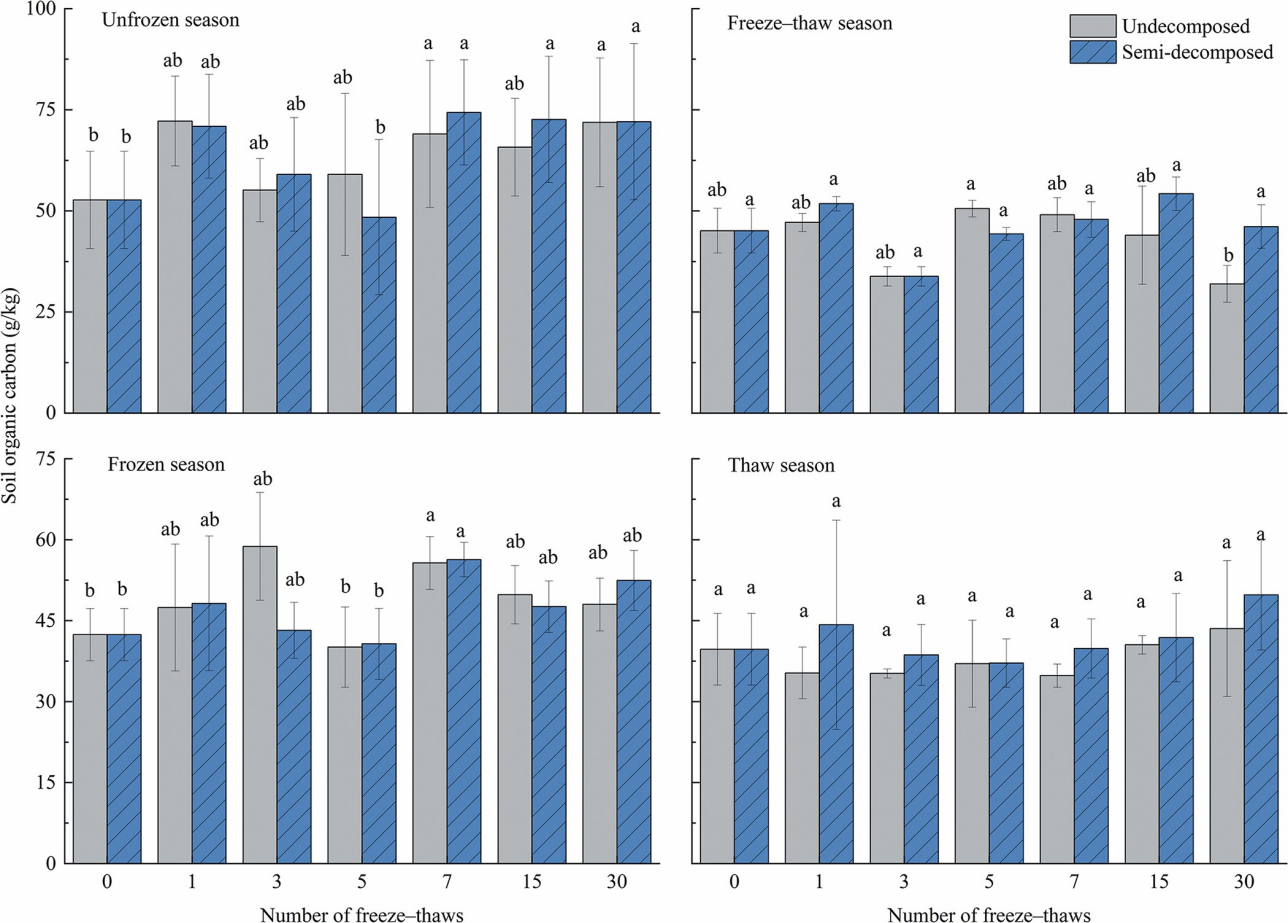
Effects of freeze–thaw time in different seasons on the soil carbon concentration at various degrees of litter decomposition. Mean ± SE is shown in the figures. Different letters indicate significant difference at 0.05 level.

The interaction between the number of freeze–thaw cycles and season had a highly significant effect on soil organic carbon (*P*<0.001). Fig 5 shows that after the 7th and 30th freeze–thaw during the unfrozen season, soil organic carbon in the undecomposed litter was significantly higher than the initial value; after the 7th, 15th, and 30th freeze–thaws, the soil organic carbon of semi-decomposed litter was considerably higher than that after the 0th and 5th freeze–thaw. During the freeze–thaw season, soil organic carbon in the undecomposed litter was significantly lower after the 30th freeze–thaw than after the 5th freeze–thaw, and the number of freeze–thaw treatments had no effect on soil organic carbon in the semi-decomposed litter. During the frozen season, organic carbon in the litter was considerably higher after the 7th freeze–thaw than before the initial value and after the 5th freeze–thaw. The number of freeze–thaw cycles during the thaw season did not affect the soil organic carbon.

**Fig 6:**
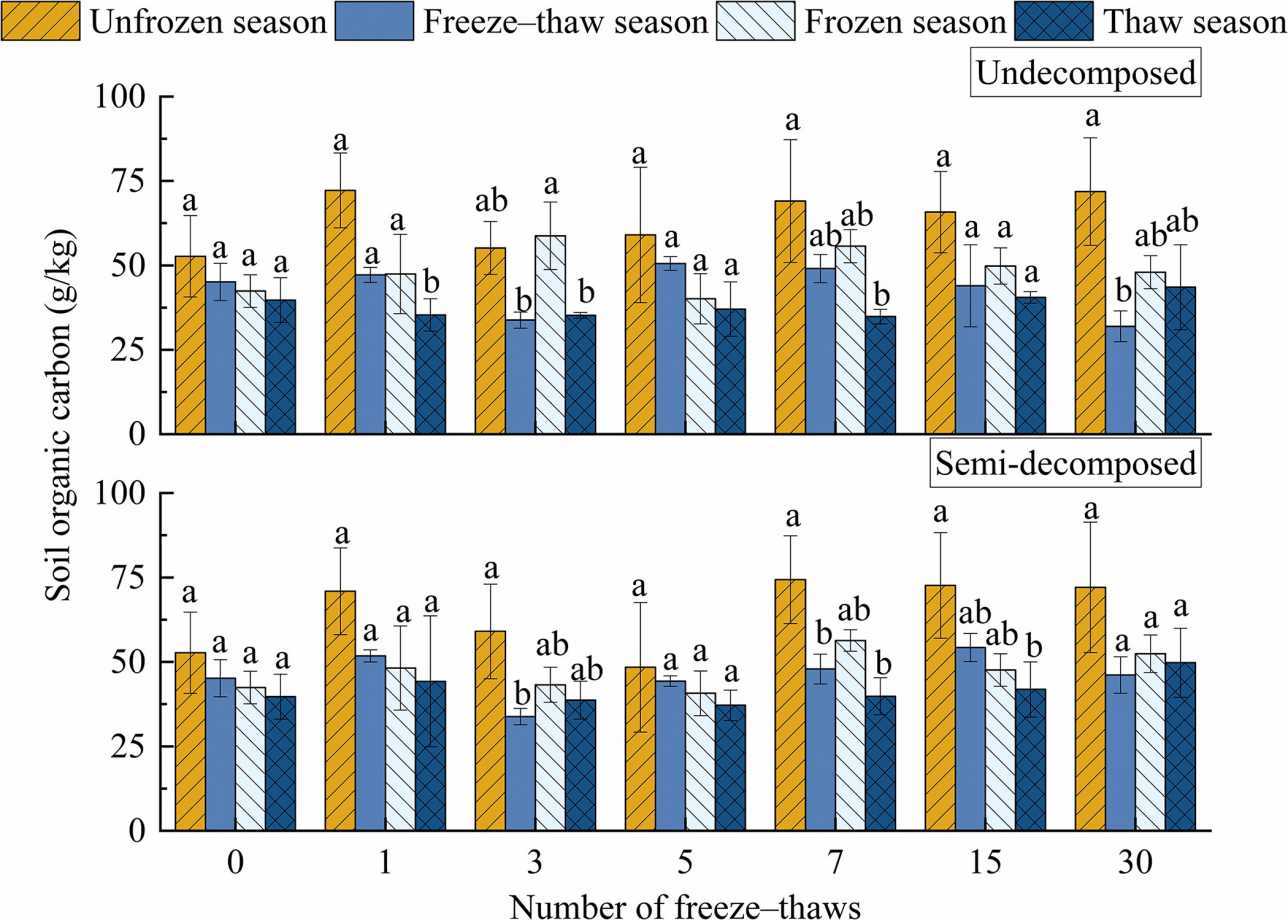
Effects of season on the carbon content of soil litter at various levels of decomposition under freeze–thaw treatment. Mean ± SE is shown in the figures. Different letters indicate significant difference at 0.05 level.

The effect of season on soil organic carbon was highly significant (*P* = 0.003). Fig 6 shows that soil organic carbon in undecomposed litter soil was significantly higher in the 1st and 7th unfrozen seasons than in the thaw season, significantly higher in the 3rd frozen season than in the freeze–thaw and thaw seasons, and significantly higher in the 30th unfrozen season than in the freeze–thaw season. Soil organic carbon was significantly higher in semi-decomposed litter soil in the 3rd unfrozen season than in the freeze–thaw season, significantly higher in the 7th unfrozen season than in the freeze–thaw and thaw seasons, and significantly higher in the 15th unfrozen season than in the thaw season. The number of freeze–thaw seasons had no effect on the organic carbon of litter.

## Discussion

### Effect of environmental factors on the decomposition of litter

Forests are the most cost-effective carbon absorbers because they fix atmospheric CO_2_ in the form of organic matter in the plant body and soil through photosynthesis, thus reducing the accumulation of greenhouse gases. Forests thus play roles in the global carbon cycle as a source, reservoir, and sink, and any increase or decrease in their carbon stocks can affect variation in atmospheric CO_2_ concentrations [34]. All organic matter created by biological components of a forest ecosystem and returned to the forest floor as a source of material and energy for decomposers to sustain ecosystem function is referred to as apoplankton. The biogeochemical cycle of forest ecosystems includes the breakdown of apoplastic matter, and the rate of decomposition of this matter has a substantial effect on the productivity of ecosystems. Litter decomposition also has a considerable effect on the physicochemical quality of soil and is a primary predictor of the biomass and nutrient content of the forest floor [35]. Forest litter is an important component of the biomass and material cycle of forest ecosystems, as it releases nutrients and energy fixed by forest plants from the soil and atmosphere to the environment through microbial decomposition. It is thus a major link in the energy flow and material cycle of forest ecosystems and one of the most important media for carbon exchange between the atmosphere and soil [36]. The forest floor is subjected to long periods of seasonal freezing and thawing at high latitudes and altitudes, which affects the breakdown of apoplastic material by mechanical fragmentation, the leaching of the material’s physical structure, as well as the leaching of biological constituents [37,38]. The frequency of freeze–thaw cycles has a substantial effect on the composition of litter. The number of freeze–thaw cycles had a substantial effect on the mass loss of litter (*P*<0.05). The rate of the mass loss of litter was high from the start of the 1st to the 7th freeze–thaw in the unfrozen season (Figs 1 and 2), indicating that the residual rate was much lower after the 7th freeze–thaw than the initial value. The rate of mass loss of semi-decomposing litter decreased, increased, and then decreased, and the rate of mass loss peaked after the 7th freeze–thaw; the residual rate of semi-decomposing litter was significantly lower after the 7th freeze–thaw than its initial value.

Because of the higher temperatures and greater mass-loss rates in the unfrozen season, the overall rate of mass loss was higher in the unfrozen season than in the other three seasons. Because the temperature fluctuates above and below 0°C during the freeze–thaw season, the overall rate of mass loss of undecomposed litter was higher than that of semi-decomposed litter. The physical fragmentation of undecomposed litter during the freeze–thaw season enhanced the rate of mass loss of undecomposed litter. The effect of freeze–thaws on the semi-decomposed litter was minimal. The rate of mass loss was not lower in the frozen season than in other seasons. In the frozen season, the residual rate was much lower after the 5th and 15th freeze–thaw cycles than its initial value. This suggests that mass loss still occurs during the frozen season. The lowest rate of mass loss was observed during the thaw season. The number of freeze–thaw treatments did not affect the litter mass retention rate throughout the thaw season. The litter present in the thaw season did not decompose in both the freeze–thaw and frozen seasons; in other words, the litter present in the thaw season was litter that does not easily decompose. Alternatively, the water in the litter was damaged by the low temperature during the frozen season. Fluctuations in temperature above and below 0°C throughout the thaw season are insufficient for disintegrating the leaves. It has been reported that showing the rate of decomposition of broadleaf forest litter is highest in the early phases of decomposition and gradually decreases thereafter [39].

### Effect of environmental factors on litter organic carbon

Apoplankton is a vital component of ecosystems, and characterizing the volume of apoplankton and its effect on nutrient dynamics is important for understanding the material cycles and energy flows of ecosystems [40,41]. Some of the carbon in decomposing apoplankton is directly released into the atmosphere, whereas the rest enters the soil, becomes part of the soil carbon cycle, or is released into the atmosphere via soil respiration [36]. The pace of apoplastic matter decomposition and conversion reflects the rate of nutrient return and cycling on the forest floor [11]. The release of nutrients from decaying litter is primarily controlled by biotic and abiotic processes and includes leaching-enrichment-release, enrichment-release, and direct release [42]. Increasing temperatures have a substantial impact on the structure, function, dynamics, and distribution of forest vegetation, and these changes can affect the carbon sequestration capacity of forest vegetation [43–46]. The significance of temperature regulation is intimately linked to the carbon cycle processes associated with the buildup and breakdown of apoplankton [2]. Climate change has direct and indirect effects on the decomposition process [47,48] and is the most important ecological element controlling apoplankton decomposition [49]. Carbon and nutrient cycling in cold locations is affected by the mass loss and nutrient release of forest litter during the freeze–thaw season [50]. Season, the number of freeze–thaw cycles, as well as their interaction had significant effects on the organic carbon in litter (*P*<0.01). The organic carbon of litter increased at the end of the culture period in the unfrozen season and the frozen and thaw seasons; the organic carbon of the undecomposed dead litter decreased at the end of the culture period in the freeze–thaw season; and the organic carbon of the semi-decomposed dead litter increased at the end of the culture period in the freeze–thaw season. Organic carbon was higher later in the freeze–thaw stage of the unfrozen season compared with earlier in that season. After the 7th freeze–thaw in the freeze–thaw season, the organic carbon of undecomposed litter began to increase; the organic carbon of semi-decomposed litter gradually increased after the 1st freeze–thaw. The organic carbon of semi-decomposed litter in the 30th freeze–thaw cycle was substantially higher than that in the 1st freeze-thaw cycle; the number of freeze–thaws had no effect on the organic carbon of litter in the frozen season. The organic carbon of semi-decomposed litter was significantly lower than the initial value after the 5th freeze–thaw in the thaw season, which might stem from the state of decomposition of the litter and the presence of several carbon elements that had not yet been released into the soil. The number of freeze–thaws had no effect on the organic carbon of undecomposed litter in the thaw season.

The organic carbon of the litter was significantly affected by the interaction between the degree of litter decomposition and season (*P* = 0.001). The organic carbon of undecomposed dead litter was highest in the thaw season, followed by the freeze–thaw season, frozen season, and unfrozen season, and the organic carbon of semi-decomposed dead litter was highest in the frozen season, followed by the thaw season, freeze–thaw season, and unfrozen season. The organic carbon of the litter began to fix carbon at the beginning of the unfrozen season, and organic carbon increased in the freeze–thaw season. After the freeze–thaw season, the organic carbon of the undecomposed litter decreased, and the organic carbon of the semi-decomposed litter increased. The carbon of the undecomposed litter was transferred to the semi-decomposed litter, and this caused the organic carbon of the undecomposed litter to decrease in the frozen season. The soil organic carbon began to rise during the thaw season, given that the carbon of semi-decomposed litter carbon was released into the soil. In conclusion, the carbon stock of dead litter was lowest during the unfrozen season. It has been reported that litter carbon stocks are lower in the summer [51], and freeze–thaws promote the accumulation of organic carbon in the residual litter [20].

### Effect of environmental factors on soil organic carbon

In terrestrial ecosystems, the soil is the largest carbon pool, but its turnover is slow. The organic and inorganic carbon pools, as well as a small part of the soil inorganic carbon pool, comprise this system [52]. Soil organic carbon is an important component of the carbon pools of terrestrial ecosystems, and slight changes in soil organic carbon can have substantial effects on atmospheric greenhouse gas concentrations [53]. Approximately 1400–1500 Gt of carbon is stored in organic form in soils around the world, which is two to three times greater than the carbon pool in terrestrial vegetation (500–600 Gt) and more than twice as much as the carbon pool in the atmosphere (750 Gt) [54]. Forest soil contains approximately 40% of the global terrestrial soil organic carbon [55]. The amount of forest soil carbon is determined by the relationship between the amount of biomass input, the amount of carbon released by decomposition, and the amount of carbon lost into the water system; it is derived from the input of aboveground and belowground apoplastic material and the decomposition of organic matter and is in the form of decomposed plant and animal remains, debris, or organic matter [56]. As a result, the size of the soil carbon pool is affected not only by vegetation but also by local climatic conditions [57]. Climate change is linked to the decomposition and buildup of organic carbon in forest soils [58]. The accumulation of soil organic carbon is affected by various factors, including climate. Climatic conditions determine the type of vegetation, its productivity, and thus the amount of organic carbon input to the soil. Microorganisms are the primary drivers of soil organic carbon decomposition and turnover, and climate can affect the decomposition and transformation of organic carbon by microorganisms by altering soil moisture, soil aeration, and temperature [59–62]. Freeze–thaws are a common natural phenomenon in which soil freezes and thaws as a result of variation in temperature [63]. They are particularly common in the northern hemisphere, where temperatures fluctuate around 0°C; these freezing and thawing conditions affect the physical, chemical, and biological properties of the soil. In Northeast China, freeze–thaw cycles occur for approximately six months a year and thus have a substantial effect on soil organic carbon turnover [64,65]. The release of organic carbon from the soil is also accelerated by the melting of permafrost due to global warming. The depth and duration of the thaw determine the stability of the soil organic carbon pool during freeze–thaw conditions [66]. The physical breakdown of apoplankton increases with the number of freeze–thaw cycles, which promotes apoplankton decomposition. Apoplankton is an important component of the forest ecosystem carbon pool, and its decomposition plays a critical role in the formation of soil organic matter and the forest carbon cycle [2,67]. The number of seasons and freeze–thaw cycles, as well as their interaction, had a substantial effect on the soil organic carbon content in litter at different levels of decomposition (*P*<0.01). Soil organic carbon accumulates after the end of the unfrozen season due to photosynthesis, the high carbon sequestration ability of plants, and carbon fixation in the soil, and organic carbon was higher at this point compared with the initial value in the freeze–thaw phase (after the 7th freeze–thaw). During the freeze–thaw season, the organic carbon of undecomposed litter soil decreased, and the organic carbon of semi-decomposed litter slightly increased; the organic carbon of undecomposed litter soil was the highest in the 5th freeze–thaw season (50.6±2.0 g/kg), and the organic carbon of undecomposed litter soil was significantly lower after 30 freeze–thaws (31.9±4.6 g/kg) than after the 5th freeze–thaw. This is likely explained by the release of the organic carbon of undecomposed litter into the soil through the decomposition of litter; furthermore, the carbon absorbed by the soil from the semi-decomposed litter was less than the carbon released into the atmosphere by the soil. Soil organic carbon increased in both the frozen and thaw seasons, and it was higher after the 7th frozen season compared with the initial value and the value after the 5th frozen season. This might be explained by the slow release of organic carbon from litter into the soil. This result is consistent with the organic carbon transfer process of litter described above, which reflects the migration of carbon between litter and soil over time.

Fig 6 shows that soil organic carbon levels were often higher in the unfrozen season than in the other three seasons. Soil organic carbon was considerably higher in undecomposed litter soils in the 1st, 3rd, and 7th unfrozen seasons than in the thaw season; it was also significantly higher in the 3rd and 30th unfrozen seasons than in the freeze–thaw season. The soil organic carbon of semi-decomposed soil was significantly higher in the 3rd and 7th unfrozen seasons than in the freeze–thaw season; it was also significantly higher in the 7th and 15th unfrozen seasons than in the thaw season. Soil organic carbon was highest in the unfrozen season, freeze–thaw season, frozen season, thaw season to unfrozen season, frozen season, thaw season, and freeze–thaw season after 30 freeze–thaw cycles. This might stem from the fact that plants have a greater potential to sequester carbon during the unfrozen season, which is consistent with the lower organic carbon in the unfrozen season compared with the other three seasons. The progressive degradation of undecomposed litter into the semi-decomposed litter, where organic carbon is retained in the litter layer and little carbon is released into the soil, might explain the decrease in soil organic carbon during the freeze–thaw season. The increase in soil organic carbon during the frozen season most likely stems from the increase in organic carbon in the litter and lower CO_2_ evaporation, which results in a faster rate of carbon migration into the soil. Because the initial organic carbon value is lower and more carbon is trapped in the litter, the organic carbon was often lower in the thaw season than in the other three seasons. It has been reported that organic carbon levels decrease from January to March and are significantly higher in subsequent seasons [68].

The temperature was higher, the mass-loss rate was faster, and the overall mass-loss rate of litter was higher in the unfrozen season than in the other three seasons. This resulted in greater litter organic carbon and soil organic carbon in the unfrozen season because of the strong carbon sequestration capacity of plants. The temperature fluctuates above and below 0°C during the freeze–thaw season, which physically breaks the undecomposed litter and speeds up its mass-loss rate. This results in increases in the organic carbon of undecomposed litter and decreases in the soil organic carbon of undecomposed litter; the opposite patterns were observed for the organic carbon of semi-decomposed litter and soil. Some mass loss of litter was observed during the frozen season. The mass-loss rate of litter during the thaw season was the lowest; litter organic carbon decreased and soil organic carbon increased in both seasons. The organic carbon of undecomposed litter was highest in the thaw season, followed by the freeze–thaw season, frozen season, and unfrozen season; the organic carbon of semi-decomposed litter was highest in the frozen season, followed by the thaw season, freeze–thaw season, unfrozen season to freeze–thaw season, frozen season, thaw season, and unfrozen season. After freeze–thaw treatment, soil organic carbon was highest in the unfrozen season, freeze–thaw season, frozen season, thaw season to unfrozen season, frozen season, thaw season, and freeze–thaw season.

## Conclusions

The rate of litter loss is the fastest in the unfrozen season, and there is still litter mass loss in the frozen season, and the litter is not easily decomposed in the freeze-thaw and thaw seasons. The organic carbon of undecomposed litter was gradually accumulated in the freeze-thaw season. After the freeze–thaw season, the organic carbon of the un-decomposed litter decreased, and the organic carbon of the semi-decomposed litter increased. The carbon of the undecomposed litter was transferred to the semi-decomposed litter, and this caused the organic carbon of the undecomposed litter to decrease in the frozen season. The soil organic carbon began to rise during the thaw season, given that the carbon of semi-decomposed litter carbon was released into the soil.

## Supporting information

S1 File (XLSX)

S2 File (XLSX)

S3 File (XLSX)
